# Effect of cyclic meditation on anxiety and sleep quality in sailors on merchant ships—A quasi-experimental study

**DOI:** 10.3389/fpubh.2024.1363750

**Published:** 2024-07-15

**Authors:** Sukesh Paranthatta, Titty George, H. M. Vinaya, P. S. Swathi, Mangesh Pandey, Balaram Pradhan, Natesh Babu, Apar Avinash Saoji

**Affiliations:** Swami Vivekananda Yoga Anusandhana Samsthana, Bangalore, Karnataka, India

**Keywords:** yoga, shipping, occupational health, meditation, sleep, psychological health

## Abstract

**Background:**

Sailors undergo anxiety and sleep disturbances due to prolonged journeys and the nature of their work on ships. Earlier studies indicate Cyclic Meditation (CM) being beneficial for managing anxiety and improving sleep quality. Thus, the current study was designed to investigate the effect of CM on anxiety and sleep quality among sailors.

**Materials and methods:**

Fifty sailors were assigned to experimental (*n* = 25) and control (*n* = 25) groups. The experimental group received 45 min of CM, 7 days a week for 3 weeks. Control group continued with their routine activities and were offered CM practice the following 3 weeks. Hamilton Anxiety Scale for anxiety (HAM-A) and the Pittsburg Sleep Quality Index (PSQI) for sleep along with blood pressure and pulse rate were taken at baseline and by the end of 3 weeks. Data were analyzed using Repeated Measures Analysis of Variance (RM ANOVA) for within and between group effects.

**Results:**

Significant differences were found between the groups following 3 weeks for all the variables. Experimental group demonstrated reduced anxiety (*p* < 0.001) and improved sleep (*p* < 0.001) along with improvements in blood pressure and pulse rate. The control group did not show any significant changes following 3 weeks.

**Conclusion:**

CM could be incorporated as a routine for sailors to manage their anxiety and improve sleep quality during the period on board ships.

## 1 Introduction

Currently, 80% of the world trade is carried out by the international shipping industry (1). Waterborne vessels like barges, ships, boats, and sailboats are the only means of transport over the water bodies. The work of a sailor is to foresee the dangers and obstructions in the path of water-based vessels and manage cargo onboard ships and other vessels. According to the International Chamber of Shipping, the estimated worldwide population of seafarers serving on internationally trading merchant ships is 1,892,720. Of these, 857,540 are officers and 1,035,180 are ratings. The largest suppliers of ratings and officers working on merchant ships are the Philippines, the Russian Federation, Indonesia, China, and India (2).

The shipboard environment and nature of shipping services can adversely impact physical and psychological health of the onboard personnel (3). A study conducted on over 1,000 sailors reported greater concerns over habitability (e.g., high noise, high temperature, extreme weathers, shaking movements etc.) were directly associated with poorer mood and greater symptoms of anxiety and depression (4). Furthermore, these sailors often work at rotating shifts, which itself can lead to increased stress and psychopathologies (5). Additionally, numerous studies have documented the adverse impact of these environment and occupational factors on sleep health and functioning (2, 4, 6), which have in turn impact on the psychological health (7). In addition, social factors such as separation from family and friends repeatedly and for prolonged duration, with limited communication due to technological constraints, and work schedules contribute to poor mental health (3, 8). These disruptions in communication and concerns about friends and family back home can lead to loneliness or greater deployment stress (9, 10). Also, interpersonal conflict in ships can lead to stress and psychological health problems among sailors (3). Thus, anxiety and poor sleep quality are common health issues among the sailors on board.

Several coping strategies are deployed to help sailors get through the difficult working environment. Team building activities bring cohesion among sailors, which can help them stay motivated and protect each other during their time on board (11, 12). To overcome or maintain their psychological health, many ships implement mental health programs, basic mental health facilities and access to counseling services for those who need them (13, 14). Several psychotherapy methodologies including cognitive behavioral therapy are employed to combat the mental health issues among sailors (15). In addition, physical and social programs like fitness facilities and movie screenings etc. are also encouraged while on board (16). Also participating in sports can boost mental health (17). The World Health Organization and National health agencies are providing guidelines related healthcare provision in ships, including the use of alternative and complementary medical practices (18). This expansion provides a great opportunity to demonstrate the usefulness of integrative health at the healthcare system level in various fields (19). The Taheri study, along with research by Camacho-Montaño LR (20) and Williams BA (21), highlights how confinement leads to decreased physical activity and poorer sleep quality among athletes, children, and prisoners, respectively. These findings underscore the detrimental impact of confinement on various occupational groups, contributing to adverse health outcomes within the population (22).

Yoga represents a holistic approach that encompasses multiple dimensions, combining physical, mental, social, and spiritual elements within its ambit. The beneficial effects of yoga have been evidenced across diverse occupational fields. In recent studies, yoga practices are found to be useful in managing pain (23), chronic venous insufficiency and fatigue (24), visual strain (25, 26) among various occupational groups. Specific yoga practices such as meditations have been found specifically for improving sleep as well as anxiety (27–29). The practice of laughter yoga has been effective in managing sleep disturbances in a recent study (30). Mindfulness-based meditation has been useful in reducing anxiety in various study populations (31, 32). The approach to practice of meditation has been diverse and various schools have evolved different types of meditation techniques.

Cyclic Meditation (CM) is one of the meditation techniques evolved based on the principle from the Mandukya Upanishad, one of the oldest texts in Indian wisdom (33). CM as a dynamic meditation technique is performed to master the mind, offering a pathway when thoughts overwhelm the mind. The CM practice involves cyclic stimulation through asanas and relaxation along with body awareness. Earlier studies of CM have shown significant improvement in the reduction of state anxiety and improvement in memory (34), induces a state of low physiological activation, reducing anxiety, decreasing stress, enhancing attention, and discriminative abilities, and promoting better sleep with increased slow-wave sleep (35, 36).

Numerous scientific studies have revealed that the mental well-being of sailors is at risk due to the inherently stressful and hostile environment of seafaring, which has been on the rise in recent years. Given that working and leisure time are spent in the same confined environment for prolonged periods, many stressors in seafaring can be considered chronic. Since they struggle with various mental conflicts, the practice of cyclic meditation proves beneficial in addressing these challenges. However, limited studies exist on this topic, underscoring the need for this study.

## 2 Materials and methods

Trial Design and Participants: A matched controlled study with a quasi-experimental design comprising two groups (experimental and control) was executed. A total of 72 male navy sailors from the merchant ships of Wedyan and Aslaf were briefed about the study protocol. The inclusion criteria of the study included volunteers who scored more than 18 on the pre-HAM A scale, which is considered moderate anxiety and severe, and who scored more than 5 in pre-PSQI, which is associated with poor sleep quality. We included participants with no prior experience with any type of Yoga practice. We excluded volunteers who are under medication and have any disease like the presence of cognitive or neurological disorders, respiratory or cardiac, and sensory abnormalities. Finally, 54 male navy sailors with their mean (±SD) age 37.74 (9.79) years were recruited to the study. The demographic data is depicted in Table 1. A quasi-experimental, non-randomized, matched control group was used. The participants were matched based on their, age, gender and rank, and then assigned to experimental or control groups. Both groups are taken from different ships namely Wedyan and Aslaf.

**Table 1:** Demographic data comparing the baseline characteristics of the study participants.

Nationality	Experimental group (*n* = 25)	Control group (*n* = 25)	*p*-value
Filipino	15	19	0.326
Indian	7	3	
Jordanian	1	0	
Montenegrin	1	0	
Saudi Arabian	0	1	
Bulgarian	1	1	
Russian	0	1	
Ranking			
Officer	6	10	0.225
Rating	19	15	
AGE	37.44 (9.7)	38.40(10.4)	0.833
HAM_A	10.60 (7.8)	11.00(6.7)	0.846
PSQI	9.20 (2.3)	9.04(2.9)	0.829

Nominal data were compared using the Chi-square test, and ordinal and ratio data were compared using an independent sample *t*-test.

**Figure 1:**
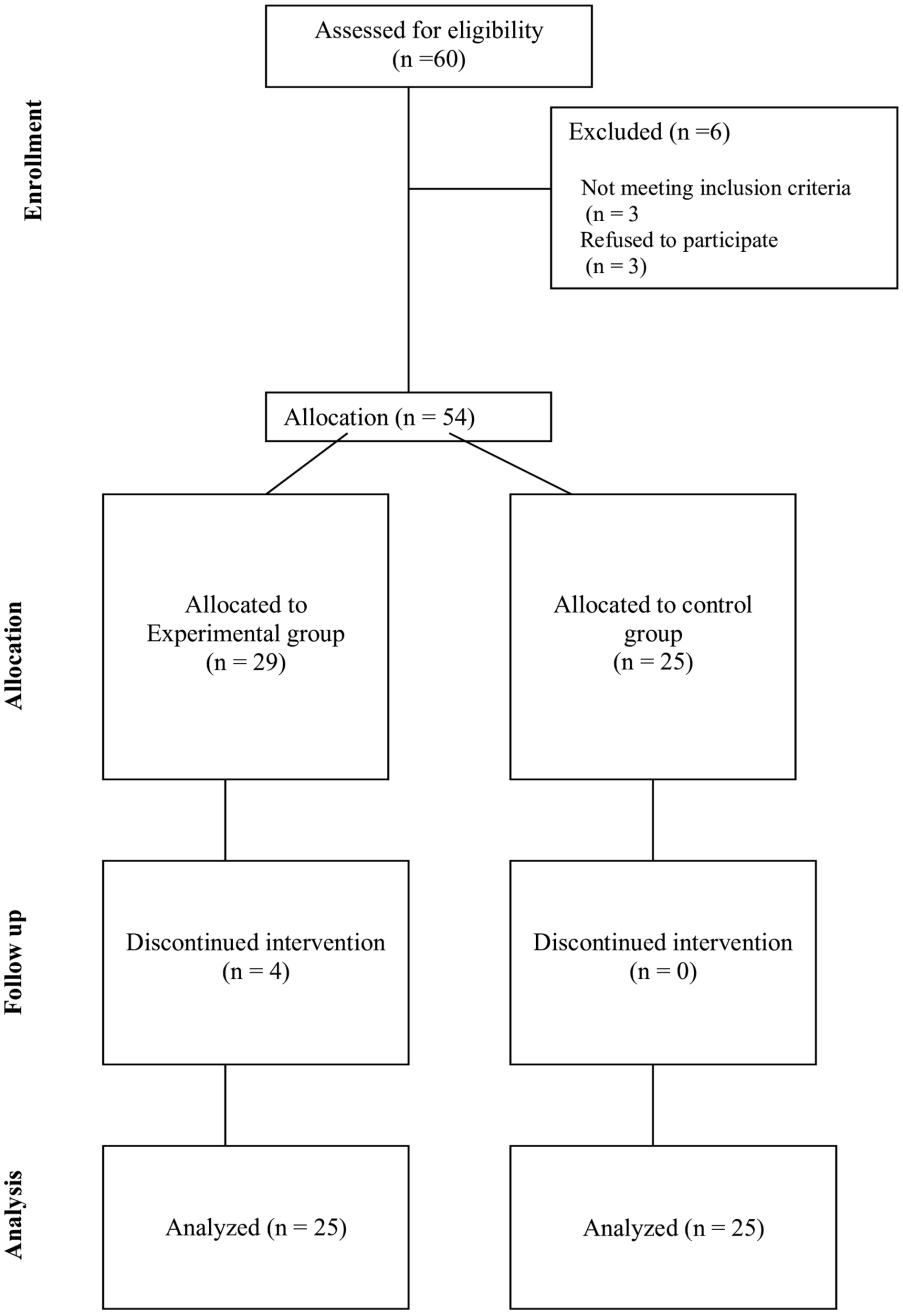
Design of the study.

Ethical consideration: The Institutional Ethics Committee of the University approved the study. Clearance was obtained from the authorities of merchant ships of Wedyan and Aslaf to conduct the meditation sessions on board and written informed consent was obtained from individual participants before their recruitment to the study. We have ensured voluntary participation and we considered their schedules and privacy (Figure 1).

### 2.1 Intervention

Experimental: Cyclic meditation: Throughout the practice, subjects kept their eyes closed, and followed pre-recorded instructions. The instructions emphasized carrying out the practice slowly, with awareness and relaxation. The practice began by repeating a verse (40 s) from the yoga text, the Mandukya Upanishad (33); followed by stimulation and relaxation of the body. The practice lasted for a total of 45 min, with the intervention administered continuously for 21 days, keeping a gap of 90 min following dinner, starting from 8:30 PM and concluding at 9:15 PM each evening.

Cyclic Meditation is a structured procedure that alternates between stimulation and relaxation techniques. Participants begin by starting prayer for 2 min and lying down in a supine position, where they experience instant relaxation for 2 min. During this phase, all body parts are gently stretched and contracted simultaneously, aiding in calming the mind. Following this, the participant transitions to lying on their left lateral side, practicing linear awareness for a period. Then, they stand in Tadasana, balancing their weight evenly on both feet (centering) for 2 min. Next, participants perform asanas such as Ardha Kati Chakrasana on both sides, focusing on slow and steady movements while observing all sensations throughout the body for 4 min on each side. After the asana practice, the participant returns to Shavasana. A quick relaxation technique is applied, focusing on abdominal movements and synchronization, followed by chanting ‘aa’ for 5 min. They then turn to the left lateral side and come to a sitting position with their legs stretched out for 2 min, followed by practicing Vajrasana, Ardha Ushtrasana, and Shashanka Asana, each for 2 min. The session concludes with the participant lying on their back in Shavasana for a deep relaxation technique. During this practice, the entire body is relaxed part by part, starting from the toes up to the head, with chanting of ‘aa’, ‘uu’, ‘mm’, and ‘om’ for 10 min. Afterward, a positive affirmation is repeated for 2 min, followed by a closing prayer for 3 min (37). The brief outline intervention is given in Table 2.

**Table 2:** Steps in cyclic meditation.

Steps	Particulars	Duration	
Step 1	**Starting Prayer**: *laye sambodhayet chittam vikShiptam shamayet punah* *sakashayam vijaaniiyaat samapraaptam na chaalayet* // **Meaning**: Stimulate and awaken the sleeping mind, calm down the distractions, recognize the innate stagnations and stay in steadiness without disturbing it. **Source**: Mandukya Karika (commentary on Mandukya Upanishad, one of the 10 principal Upanishads [part of Vedas])	02 min	
Step 2	**Instant relaxation technique (IRT)**: Lying down in supine position During this phase, all body 2 parts are gently stretched and contracted simultaneously, aiding in calming the mind. Following 3 this, the participant transitions to lying on their left lateral side, practicing linear awareness for 4 a period	02 min	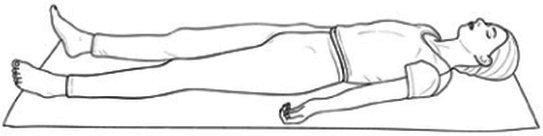 IRT in Shavasana
Step 3	**Standing Asanas** **Tadasana**: balancing their weight evenly on both feet (centering) for 2 min **Ardha Kati Chakrasana**: focusing on slow and steady movements while observing all sensations throughout the body for 4 min on each side.	10 min 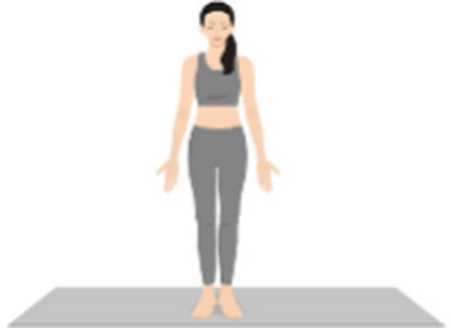 Tadasana	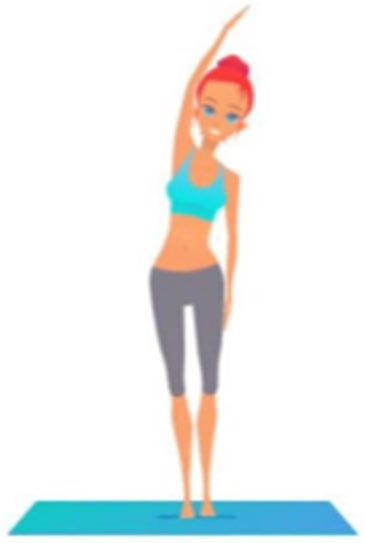 Ardha Kati Chakrasana
Step 4	**Quick relaxation technique (QRT)**: Focusing on abdominal movements and synchronization, followed by chanting ‘aa’. 5 min	05 min	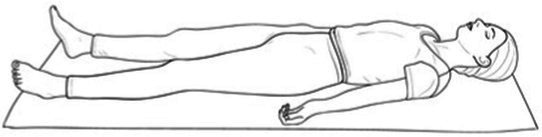 QRT in Shavasana
Step 5	**Sitting Asanas** **Dandasana**: sitting position with their legs stretched out for 2 min **Vajrasana**: is sitting posture, sitting on the heels, and placing the knees, legs, feet together. Keeping the back straight and place the palms over the thighs. Focus on the breath. Total duration of 2 min. **Shashankasana**: from the vajrasana, lift your hands straight up and bend toward the ground and stretch the hands forward and touch the ground. Total duration of 2 min. **Ardha-ustrasana**: From the vajrasana, stand on the knees and place the hands on the waist finger pointing downwards, now bend the trunk and head backwards. Remain in the posture for half a min. Return to vajrasana and relax. Total duration of 2 min.	08 min 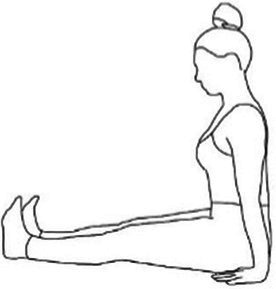 Dandasana 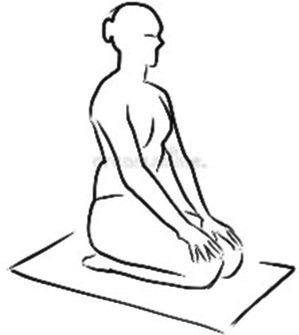 Vajrasana	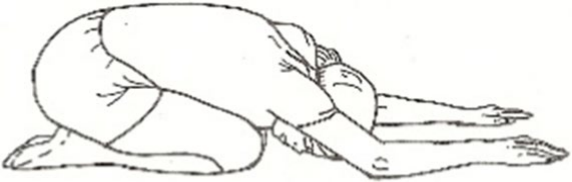 Shashankasana 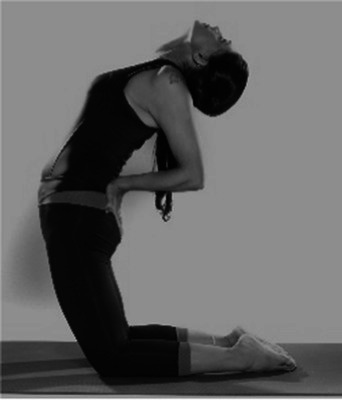 Ardha Ustrasana
Step 6	**Deep relaxation technique (DRT)**: During this practice, the entire body is relaxed part by part, starting from the toes up to the head, with chanting of ‘aa’, ‘uu’, ‘mm’, and ‘om’ for 10 min	10 min	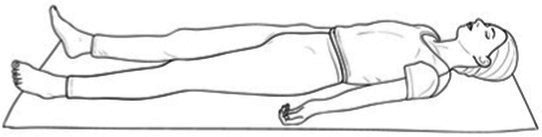 DRT in Shavasana
Step 7	**Positive Resolve**: positive resolution aligns with the concept of “sankalpa” or a positive resolve.	05 min	
Step 8	**Closing Prayer**: *Sarve bhavantu sukhinaH sarve santu niraamayaaH* *sarve bhadraaNi pashyantu maa kashchidduHkhabhaagbhaveta* *Om shaantiH shaantiH shaantiH* **Meaning**: May all be happy. May all be free from all diseases. May all see goodness and auspiciousness in everything. May none be unhappy or distressed. Om peace peace peace. **Source**: Brihadaranyaka Upanishad one of the 10 principal Upanishads (part of Vedas)	03 min	

Waitlist Control Group: The control group participants were asked to continue their routine daily activities for the follow-up duration of 21 days. Following the 21 days, they were offered the same intervention as cyclic meditation.

Assessments: The impact of cyclic meditation was assessed using self-report measures such as the Hamilton Anxiety Scale (HAM-A) for anxiety and the Pittsburg Sleep Quality Index (PSQI) for sleep. The assessment was conducted on the first day at the same time of the day, before the intervention, for both the experimental and control groups.

HAM-A assesses the presence and degree of severity of types of anxiety symptoms to provide a patient’s status ranging from mild to severe. The scale, developed by Hamilton in 1959 the scale contains 14 entries. It uses the 5-level rating method of 0 to 4 points. The total score is 0 ∼ 56, and the anxiety level can be divided as follows: < 7 means no anxiety, 7 ∼ 14 means possible anxiety, 15 ∼ 21 means certain anxiety, 21 ∼ 29 means obvious anxiety, and > 29 means severe anxiety. Score 14 is generally the critical value. The Cronbach’s α of the Hamilton Anxiety Scale was 0.77 to 0.92 (38, 39).

PSQI which is a validated questionnaire for the assessment of sleep patterns was developed by the University of Pittsburgh. It is a self-reported questionnaire that assesses sleep quality over a 1-month time interval, and addresses 7 components, i.e., subjective sleep quality, latency, duration, efficiency, disturbance, need for medications to sleep and day dysfunction due to sleepiness, with a maximum score being 21 points. A cut-off of five was considered to identify respondents with poor sleep quality. The Cronbach’s α of the PSQI fluctuated between 0.70 and 0.85 (40, 41).

Evaluation of BP and PR: We evaluated the patient’s BP and PR using an automated Oscillometric upper-arm BP monitoring device (Omron HEM7120). The participants were seated in a quiet room at a comfortable temperature and were instructed to avoid talking during the procedure. The BP measurements were started after the participants had rested for 5 min. The participants sat on a chair with their legs uncrossed and their feet flat on the floor. All BP measurements were performed on the participant’s left arm at the level of the heart and have taken measurements at the same time. The BP and PR were recorded thrice in a single sitting and the average of the three recordings was taken to ensure reliability of the data.

Data Extraction and Analysis: The data for HAM-A and PSQI were extracted using questionnaire manuals and organized in Microsoft Excel version 2016. The BP was organized in systolic and diastolic BP and expressed as mmHg. PR was expressed in beats/min. Data analysis was performed using the JASP statistical package version 0.17.0 (42).

## 3 Results

A total of 54 participants took part in the study. Four participants were lost to follow-up in an experimental study, whose data were not considered for final analysis. Finally, the data of 50 participants were analyzed. The demographic data were compared using the Chi-square test, and ordinal and ratio data were compared using RM ANOVA. The demographic data is represented in Table 1.

### 3.1 Within-group changes

The RM ANOVA demonstrated significant within-subjects in HAM-A scores *F* = 18.653, *p* < 0.001; PSQI scores *F* = 33.391, *p* < 0.001, Systolic BP *F* = 8.231, *p* = 0.006, Diastolic BP *F* = 13.582, *p* < 0.001, Mean arterial pressure *F* = 19.757, *p* < 0.001, Pulse rate *F* = 10.109, *p* = 0.003. The within-subjects changes obtained through RM ANOVA are presented in Table 3.

**Table 3:** Results of the repeated measures analysis of variance (RM-ANOVA) for within-subject effects.

Variable	Time (df = 1.48)			Time*Group (df = 1.48)		
	F	*p*	η^2^_p_	F	*p*	η^2^_p_
HAM-A Scores	18.653	<0.001	0.280	21.782	< 0.001	0.312
PSQI Scores	33.391	<0.001	0.410	33.391	<0.001	0.410
Systolic Blood Pressure (mmHg)	8.231	=0.006	0.146	10.750	=0.002	0.183
Diastolic Blood Pressure (mmHg)	13.582	<0.001	0.221	10.790	=0.002	0.184
Mean Arterial Pressure (mmHg)	19.757	<0.001	0.292	19.992	<0.001	0.294
Pulse Rate (beats/min)	10.109	=0.003	0.174	9.452	=0.003	0.165

HAM-A, Hamilton Anxiety Rating Scale; PSQI, Pittsburgh Sleep Quality Inventory.

### 3.2 Between-group changes

Pairwise comparisons between the groups performed through RM ANOVA with Holms corrections demonstrated significantly higher scores following cyclic meditation when compared with baseline for HAM-A scores, *t* = − 3.257, *p* = 0.009; PSQI scores, *t* = −4.682, *p* < 0.001; Systolic BP, *t* = −2.095, *p* = 0.041; Diastolic BP, *t* = −2.446, *p* = 0.018; Mean arterial pressure, *t* = 0.717, *p* = 0.015; Pulse rate, *t* = −2.037, *p* = 0.014. The effect sizes and t-values between groups using RM ANOVA with Holm’s correction along with the group mean and SD are reported in Table 4.

**Table 4:** *Post-hoc* analysis for RM-ANOVA with Holm’s corrections.

Variable	Experimental group				Control group				Between group analyses for post-values		
	Pre	Post	*t*-value	Cohen’s d	Pre	Post	*t*-value	Cohen’s d	*p*-value	*t*-value	Cohen’s d
HAM-A Scores	10.60 ± 7.77	5.44 ± 4.05***	6.364	0.825	11.00 ± 6.62	11.20 ± 5.97	−0.246	−0.032	=0.009	−3.257	−0.921
PSQI Scores	9.20 ± 2.29	6.00 ± 1.65***	8.872	1.394	9.04 ± 2.89	9.04 ± 2.16	0.000	0.000	<0.001	−4.682	−1.324
Systolic Blood Pressure (mmHg)	132.00 ± 9.73	125.40 ± 9.58***	4.347	0.690	130.48 ± 9.86	130.92 ± 9.03	−0.290	−0.046	=0.041	−2.095	−0.593
Diastolic Blood Pressure (mmHg)	82.96 ± 6.82	79.84 ± 6.17***	4.929	0.637	83.28 ± 4.31	83.08 ± 3.99	0.283	0.037	=0.018	−2.446	−0.692
Mean Arterial Pressure (mmHg)	99.31 ± 6.97	94.79 ± 6.57***	6.305	0.738	99.01 ± 5.62	99.03 ± 5.18	−0.019	−0.002	=0.015	−0.717	0.300
Pulse Rate (beats/min)	77.12 ± 5.97	72.36 ± 5.77***	4.422	0.846	75.56 ± 5.67	75.48 ± 5.03	0.074	0.014	=0.047	−2.037	−0.576

HAM-A, Hamilton Anxiety Rating Scale; PSQI, Pittsburgh Sleep Quality Inventory; ***p < 0.001 for within subject comparison.

### 3.3 Adverse effects

Throughout the study, participants were encouraged to report any adverse effects to the investigators. Only two participants in the experimental group reported mild headaches following the practice of CM on the first day. No other adverse events were reported.

## 4 Discussion

The purpose of this study is to observe the impact of 21 days of Cyclic meditation intervention on sleep quality and anxiety levels in merchant navy sailors while on board the vessel. After 21 days of CM, statistically significant results are noticed in sleep quality, anxiety levels, and secondary variables of Systolic blood pressure and Diastolic Blood pressure, mean arterial pressure and pulse rate. To the best of our knowledge, this is the first study to assess the effect of cyclic meditation on anxiety and sleep quality in merchant navy sailors.

The study outcomes are similar to earlier studies of cyclic meditation. Based on a polysomnographic study conducted on 30 male participants, it was observed that CM for 2 days resulted in a decrease in the number of rapid eye movements and awakenings at night (35). Additionally, the self-rating of sleep showed an increase in the feeling that sleep was refreshing and an improvement of sleep duration (43). The immediate effect of CM was assessed by using the Wechler Memory Scale, Spielberg’s State Anxiety Inventory, and Digit Span Test. The results showed that the CM participants had a heightened memory score, improved attention, and a significant reduction in anxiety (34). Another study conducted on the immediate effect of CM evaluated the performance of participants on the Six letter cancelation test, this test is known to assess selective attention and visual scanning abilities. These findings suggest that CM has a positive impact on selection attention & visual scanning abilities (44, 45).

A study involving 26 asymptomatic male managers revealed that a two-day CM (stress management) program led to a notable decrease in both occupational stress levels and baseline autonomic arousal. This reduction in sympathetic activity indicates a significant positive impact. The mechanism driving the decline in stress levels may involve both decreased autonomic arousal and psychological factors. Coccharia’s research (46) supports this by highlighting the effectiveness of yoga in managing stress among occupational workers. Several other studies indicate beneficial role of yoga practices in various occupational settings, including farmers (47), industry workers (23, 24), healthcare workers (48), computer professionals (26) etc. Consequently, employers are encouraged to incorporate yoga into their workplace wellness programs for its potential benefits in stress management (49).

According to epidemiological studies, 50% of individuals with anxiety suffer from sleep deprivation, particularly insomnia. This deprivation of sleep can lead to a negative impact on work efficiency. Work stress, which manifests as fatigue and frustration, is associated with various negative reactions, including job dissatisfaction, low organizational commitment, and a high propensity to resign. Ultimately, anxiety negatively impacts employee performance (50).

According to studies, practicing yoga has been shown to improve sleep and reduce anxiety levels (51), which in turn helps to reduce occupational stress and improve work efficiency (52, 53). Several nonpharmacological interventions that could help sleep disturbances have been in practice such as acupuncture and aromatherapy (54, 55). Our study’s results conform with these earlier findings from studies conducted in other work environments (56). However, this study is the first one conducted on board in a group of international seafarers, applying the intervention of Cyclic Meditation specifically. Our results of increased sleep quality and reduced levels of anxiety indicate better performance and could bring a safer work and living atmosphere on board.

Several limitations were encountered, as the study setting was on board the ships. The sample size was inevitably restricted, and collecting numerous samples simultaneously posed a challenge due to the crew working in shifts aboard the vessel. Also, the sample was restricted to male seaboard crew only, which limits the generalization of the results of this study. Additionally, the absence of an active control group could be considered another major limitation of the study. Longer interventions cannot be utilized as they may hamper the work rest hours onboard. More variables could have been used to make the study stronger.

## 5 Conclusion

The practice of 21 days of cyclic meditation led to positive changes in sleep quality and reduced anxiety in merchant navy sailors while sailing. This initial quasi-experimental study can pave the way for large-scale trials with robust designs and an active control group. Looking ahead, the long-term effects of cyclic meditation could offer valuable support in managing stressors inherent in ship life, thereby enhancing overall well-being. Additionally, there is potential to explore the impact of cyclic meditation on depression levels among sailors, offering promising avenues for future research and intervention.

## Data availability statement

The raw data supporting the conclusions of this article will be made available by the authors, without undue reservation.

## Ethics statement

The studies involving humans were approved by Swami Vivekananda Yoga Anusandhana Samsthana. The studies were conducted in accordance with the local legislation and institutional requirements. The participants provided their written informed consent to participate in this study.

## Author contributions

SP: Conceptualization, Data curation, Investigation, Project administration, Writing – original draft, Writing – review & editing. TG: Conceptualization, Data curation, Investigation, Project administration, Writing – original draft, Writing – review & editing. HV: Writing – original draft, Writing – review & editing. PS: Writing – original draft, Writing – review & editing. MP: Writing – original draft, Writing – review & editing. BP: Conceptualization, Formal analysis, Supervision, Writing – original draft, Writing – review & editing. NB: Supervision, Writing – original draft, Writing – review & editing. AS: Data curation, Formal analysis, Methodology, Software, Writing – original draft, Writing – review & editing.

## References

[1] Brodie P. International chamber of shipping. In: Commercial shipping handbook. 3rd edn, Routledge. (2015).

[2] Russell DW Markwald RR Jameson JT. Self-reported sleep and sleep deficiency: results from a large initiative of sailors attached to U.S. navy warships. J Sleep Res. (2021) 30:e13397. doi: 10.1111/jsr.13397, PMID: 34187090 PMC9285824

[3] Brooks SK Greenberg N. Mental health and psychological wellbeing of maritime personnel: a systematic review. BMC Psychol. (2022) 10:139. doi: 10.1186/S40359-022-00850-4, PMID: 35637491 PMC9150387

[4] Matsangas P Shattuck NL. Habitability in berthing compartments and well-being of sailors working on U.S. navy surface ships. Hum Factors. (2021) 63:462–73. doi: 10.1177/0018720820906050, PMID: 32109155

[5] Schmied EA Martin RM Harrison EM Perez VG Thomsen CJ. Studying the health and performance of shipboard sailors: an evidence map. Mil Med. (2021) 186:E512–24. doi: 10.1093/MILMED/USAA459, PMID: 33211097

[6] Schmied EA Harrison EM Dell’Acqua RG Perez VG Glickman G Hurtado SL. A qualitative examination of factors that influence sleep among shipboard sailors. Mil Med. (2021) 186:E160–8. doi: 10.1093/MILMED/USAA321, PMID: 33516158

[7] Harrison EM Easterling AP Schmied EA Hurtado SL Glickman GL. Chronotype and self-reported sleep, alertness, and mental health in U.S. sailors. Mil Med Res. (2021) 8:43. doi: 10.1186/S40779-021-00335-2 34376248 PMC8353852

[8] Carter SP Renshaw KD. Spousal communication during military deployments: A review. J Fam Issues. (2016) 37:2309–32. doi: 10.1177/0192513X14567956

[9] Cacioppo JT Cacioppo S Adler AB Lester PB Mcgurk D Thomas JL. The cultural context of loneliness: risk factors in active duty soldiers. J Soc Clin Psychol. (2016) 35:865–82. doi: 10.1521/JSCP.2016.35.10.865

[10] Ramchand R Rudavsky R Grant S Tanielian T Jaycox L. Prevalence of, risk factors for, and consequences of posttraumatic stress disorder and other mental health problems in military populations deployed to Iraq and Afghanistan. Curr Psychiatry Rep. (2015) 17:37. doi: 10.1007/S11920-015-0575-Z, PMID: 25876141

[11] Kanesarajah J Waller M Zheng WY Dobson AJ. Unit cohesion, traumatic exposure and mental health of military personnel. Occup Med (Lond). (2016) 66:308–15. doi: 10.1093/OCCMED/KQW009, PMID: 26874354

[12] Mcandrew LM Markowitz S Lu SE Borders A Rothman D Quigley KS. Resilience during war: better unit cohesion and reductions in avoidant coping are associated with better mental health function after combat deployment. Psychol Trauma. (2017) 9:52–61. doi: 10.1037/TRA0000152, PMID: 27455138 PMC6549499

[13] Pipere A Mārtinsone K Regzdiņa-Pelēķe L Grišķeviča I. Sailing across the Atlantic: an exploration of the psychological experience using arts-based research. Front Psychol. (2020) 11:572028. doi: 10.3389/fpsyg.2020.572028, PMID: 33162914 PMC7591676

[14] Schmied EA Harrison EM Englert RM Thomsen CJ Glassman LH. Challenges and opportunities to maximize mental health among shipboard sailors: A qualitative study. Mil Behav Health. (2023):1–12. doi: 10.1080/21635781.2023.2258785

[15] Baker JC Grover S Gunn LH Charles C Rikli H Franks MJ. Group brief cognitive behavioral therapy for suicide prevention compared to dialectal behavior therapy skills group for military service members: a study protocol of a randomized controlled trial. BMC Psychiatry. (2023) 23:904. doi: 10.1186/s12888-023-05282-x, PMID: 38053122 PMC10696749

[16] Borges GA Tortorella G Rossini M Portioli-Staudacher A. Lean implementation in healthcare supply chain: a scoping review. J Health Organ Manag. (2019) 33:304–22. doi: 10.1108/JHOM-06-2018-0176 31122116

[17] Tahira S. The association between sports participation and physical fitness. Int J Sport Stud Health. (2022) 4:127001. doi: 10.5812/intjssh-127001

[18] Rees L Weil A. Integrated medicine: imbues orthodox medicine with the values of complementary medicine. BMJ: British Med J. (2001) 322:119–20. doi: 10.1136/BMJ.322.7279.119 PMC1119398 11159553

[19] Madsen C Vaughan M Koehlmoos TP. Use of integrative medicine in the United States military health system. Evid Based Complement Alternat Med. (2017) 2017:1–11. doi: 10.1155/2017/9529257 PMC5485330 28690665

[20] Camacho-Montaño LR Iranzo A Martínez-Piédrola RM Camacho-Montaño LM Huertas-Hoyas E Serrada-Tejeda S. Effects of COVID-19 home confinement on sleep in children: A systematic review. Sleep Med Rev. (2022) 62:101596. doi: 10.1016/j.smrv.2022.101596, PMID: 35183816 PMC8810276

[21] Williams BA. Older prisoners and the physical health effects of solitary confinement. Am J Public Health. (2016) 106:2126–7. doi: 10.2105/AJPH.2016.303468, PMID: 27831779 PMC5105008

[22] Taheri M Irandoust K Reynoso-Sánchez LF Muñoz-Helú H Cruz-Morales KN Torres-Ramírez R. Effects of home confinement on physical activity, nutrition, and sleep quality during the COVID-19 outbreak in amateur and elite athletes. Front Nutr. (2023) 10:1143340. doi: 10.3389/fnut.2023.1143340, PMID: 37139442 PMC10150803

[23] Pravalika B Yamuna U Saoji AA. Yoga for musculoskeletal pain, discomfort, perceived stress, and quality of sleep in industry workers: a randomized controlled trial. Int Arch Occup Environ Health. (2023) 96:1349–60. doi: 10.1007/S00420-023-02013-3, PMID: 37821618

[24] Yamuna U Pravalika B Madle K Majumdar V Saoji AA. Effect of yoga in industrial workers with chronic venous insufficiency: A randomized controlled trial. J Integrative Complement Med. (2024) 96:1349–60. doi: 10.1089/jicm.2023.0691, PMID: 38484315

[25] Saoji AA Swathi PS Bhat R Bansal B Mohanty S Raj Lakshmi RKR. Exploring the effect of Trataka (A yogic cleansing technique) and cold eye pack on eye strain during COVID-19 pandemic: A randomized three-arm trial. J Integrative Complement Med. (2023) 30:345–51. doi: 10.1089/JICM.2023.0175, PMID: 37852005

[26] Swathi PS Saoji AA Bhat R. The role of trataka in ameliorating visual strain and promoting psychological well-being during prolonged use of digital displays: A randomized controlled trial. Work. (2022) 71:327–33. doi: 10.3233/WOR-210834, PMID: 35095011

[27] Rusch HL Rosario M Levison LM Olivera A Livingston WS Wu T. The effect of mindfulness meditation on sleep quality: a systematic review and meta-analysis of randomized controlled trials. Ann N Y Acad Sci. (2019) 1445:5–16. doi: 10.1111/NYAS.13996, PMID: 30575050 PMC6557693

[28] Goyal M Singh S Sibinga EMS Gould NF Rowland-Seymour A Sharma R. Meditation programs for psychological stress and well-being: a systematic review and meta-analysis. JAMA Intern Med. (2014) 174:357–68. doi: 10.1001/JAMAINTERNMED.2013.13018, PMID: 24395196 PMC4142584

[29] Malviya S Saoji AA Pravalika B. Yoga nidra for mental health: A systematic review of current evidence. J Spiritual Ment Health. (2023):1–27. doi: 10.1080/19349637.2023.2290249

[30] Heidari E Shirazi M Sanaguye Moharer GR. The effectiveness of laughter yoga training on quality of sleep and positive and negative affect of female teachers with diabetes. Applied Family Therapy J. (2023) 4:49–68. doi: 10.61838/kman.aftj.4.4.4

[31] Mohammad Salehi S Yousefi N Moradi O. Comparing the effectiveness of Wells’ Meta-cognition training with Kabat-Zinn’s mindfulness training on academic procrastination of students with math anxiety. J Adolescent Youth Psychol Stud. (2022) 3:452–67. doi: 10.61838/kman.jayps.3.3.35

[32] Heidari M Mirshabani ZS Sadegh Masjedi A Mortezaei H. The effectiveness of mindfulness group therapy based on cognition in reducing anxiety and increasing the quality of life of couples with delinquent husbands. Applied Family Therapy J. (2023) 4:291–303. doi: 10.61838/kman.aftj.4.2.18

[33] Nagendra HR Nagaratna R. New perspectives in stress management (SMET). Bengaluru: Swami Vivekananda Yoga Prakashana (2014).

[34] Subramanya P Telles S. Effect of two yoga-based relaxation techniques on memory scores and state anxiety. Biopsychosoc Med. (2009) 3:8. doi: 10.1186/1751-0759-3-8, PMID: 19674483 PMC2734564

[35] Patra S Telles S. Positive impact of cyclic meditation on subsequent sleep. Med Sci Monit. (2009) 15:CR375–81. PMID: 19564829

[36] Patra S Telles S. Heart rate variability during sleep following the practice of cyclic meditation and supine rest. Appl Psychophysiol Biofeedback. (2010) 35:135–40. doi: 10.1007/s10484-009-9114-1, PMID: 19838801

[37] Sarang SP Telles S. Changes in p300 following two yoga-based relaxation techniques. Int J Neurosci. (2006) 116:1419–30. doi: 10.1080/00207450500514193 17145677

[38] Thompson E. Hamilton rating scale for anxiety (HAM-A). Occup Med (Chic Ill). (2015) 65:601–1. doi: 10.1093/occmed/kqv054 26370845

[39] Hamilton M. The assessment of anxiety states by rating. Br J Med Psychol. (1959) 32:50–5. doi: 10.1111/j.2044-8341.1959.tb00467.x 13638508

[40] Mollayeva T Thurairajah P Burton K Mollayeva S Shapiro CM Colantonio A. The Pittsburgh sleep quality index as a screening tool for sleep dysfunction in clinical and non-clinical samples: A systematic review and meta-analysis. Sleep Med Rev. (2016) 25:52–73. doi: 10.1016/J.SMRV.2015.01.009, PMID: 26163057

[41] Carpenter JS Andrykowski MA. Psychometric evaluation of the Pittsburgh sleep quality index. J Psychosom Res. (1998) 45:5–13. doi: 10.1016/S0022-3999(97)00298-5 9720850

[42] JASP Team. JASP version 0.14.1. (2020). Available at: https://jasp-stats.org/

[43] Subramanya P Telles S. A review of the scientific studies on cyclic meditation. Int J Yoga. (2009) 2:46–8. doi: 10.4103/0973-6131.60043, PMID: 20842263 PMC2934575

[44] Sarang SP Telles S. Immediate effect of two yoga-based relaxation techniques on performance in a letter-cancellation task. Percept Mot Skills. (2007) 105:379–85. doi: 10.2466/pms.105.2.379-385, PMID: 18065059

[45] Subramanya P Telles S. Performance on psychomotor tasks following two yoga-based relaxation techniques. Percept Mot Skills. (2009) 109:563–76. doi: 10.2466/PMS.109.2.563-576, PMID: 20038010

[46] Krylov SS Petrov AN Losev SS Georgianova EK Lychakov AV. Behavioral effects of thyroliberin on animals of various species. Farmakol Toksikol. (1986) 49:30–3. 3081364

[47] Dhansoia V Majumdar V Manjunath N Singh Gaharwar U Singh D. Breathing-focused yoga intervention on respiratory decline in chronically pesticide-exposed farmers: A randomized controlled trial. Front Med (Lausanne). (2022) 9. doi: 10.3389/fmed.2022.807612, PMID: 35372380 PMC8965718

[48] Upadhyay V Saoji AA Verma A Saxena V. Development and validation of 20-min yoga module for reducing burnout among healthcare worker(s). Complement Ther Clin Pract. (2022) 46:101543. doi: 10.1016/j.ctcp.2022.101543, PMID: 35134698

[49] Zhang M Murphy B Cabanilla A Yidi C. Physical relaxation for occupational stress in healthcare workers: A systematic review and network meta-analysis of randomized controlled trials. J Occup Health. (2021) 63:e12243. doi: 10.1002/1348-9585.12243, PMID: 34235817 PMC8263904

[50] Chen B Wang L Li B Liu W. Work stress, mental health, and employee performance. Front Psychol. (2022) 13:1006580. doi: 10.3389/fpsyg.2022.1006580, PMID: 36425815 PMC9679506

[51] Nalgirkar S Vinchurkar S Saoji A Mohanty S. Yoga as a therapeutic intervention in the Management of Dysfunctional Uterine Bleeding: A controlled pilot study. J Midlife Health. (2018) 9:8–13. doi: 10.4103/jmh.JMH_76_17 29628722 PMC5879852

[52] Strijk JE Proper KI van Mechelen W van der Beek AJ. Effectiveness of a worksite lifestyle intervention on vitality, work engagement, productivity, and sick leave: results of a randomized controlled trial. Scand J Work Environ Health. (2013) 39:66–75. doi: 10.5271/sjweh.3311, PMID: 22740100

[53] Zheng L-W Chen Y Chen F Zhang P Wu L-F. Effect of acupressure on sleep quality of middle-aged and elderly patients with hypertension. Int J Nurs Sci. (2014) 1:334–8. doi: 10.1016/j.ijnss.2014.10.012

[54] Das P Mohanty S Saoji AA. Influence of acupuncture with three specific acu-points on quality of sleep in residents of an elderly nursing home in rural India: A pilot randomized placebo-controlled trial. Adv Integr Med. (2022) 9:110–4. doi: 10.1016/j.aimed.2022.02.001

[55] Sun A Wu X. Efficacy of non-pharmacological interventions on improving sleep quality in depressed patients: A systematic review and network meta-analysis. J Psychosom Res. (2023) 172:111435. doi: 10.1016/j.jpsychores.2023.111435, PMID: 37451171

[56] Cocchiara RA Peruzzo M Mannocci A Ottolenghi L Villari P Polimeni A. The use of yoga to manage stress and burnout in healthcare workers: A systematic review. J Clin Med. (2019) 8:284. doi: 10.3390/JCM8030284, PMID: 30813641 PMC6462946

