# Corrigendum: Effect of cyclic meditation on anxiety and sleep quality in sailors on merchant ships—A quasi-experimental study

**DOI:** 10.3389/fpubh.2025.1605273

**Published:** 2025-04-16

**Authors:** Sukesh Paranthatta, Titty George, H. M. Vinaya, P. S. Swathi, Mangesh Pandey, Balaram Pradhan, Natesh Babu, Apar Avinash Saoji

**Affiliations:** Swami Vivekananda Yoga Anusandhana Samsthana, Bangalore, Karnataka, India

**Keywords:** yoga, shipping, occupational health, meditation, sleep, psychological health

In the published article, there was an error in the **Funding statement**.

The correct Funding statement appears below.

